# Prognostic factors associated with ^18^FDG-PET/CT in esophageal squamous cell carcinoma after trimodality treatment

**DOI:** 10.1186/s12885-022-09852-2

**Published:** 2022-07-14

**Authors:** Wei-Hsiang Feng, Ying-Yi Chen, Yen‐Shou Kuo, Kuan-Hsun Lin, Yuan-Ming Tsai, Ti-Hui Wu, Hsu-Kai Huang, Tsai-Wang Huang

**Affiliations:** Division of Thoracic Surgery, Department of Surgery, Tri‐Service General Hospital, National Defense Medical Center, No. 325, Sec. 2, Chenggong Rd., Neihu Dist, Taipei City 114, Taiwan

**Keywords:** Esophageal squamous cell carcinoma, ^18^FDG-PET/CT, SUVmax, Pathological complete response, Prognostic factors

## Abstract

**Purpose:**

This study aimed to determine the pathological complete response (pCR), overall survival (OS), and disease-free survival (DFS) in patients with locally advanced esophageal squamous cell carcinoma (ESCC) using post-neoadjuvant chemoradiotherapy (nCRT) F-18-fluorodeoxyglucose (^18^FDG).

**Methods:**

This is a retrospective study of patients with locally advanced ESCC receiving nCRT and then esophagectomy between January 2011 and December 2018 in the Tri-Service General Hospital, Taipei, Taiwan. A total of 50 patients were enrolled in the study. Survival analysis was performed using the Kaplan–Meier method and Cox proportional hazards model. Univariate and multivariate analysis were used to determine the independent prognostic factors.

**Results:**

Fifty patients were enrolled in the study, and 18 had pathological complete response. Post-nCRT SUVmax ≥ 3 is a poor prognostic factor associated with overall survival (HR: 3.665, *P* = 0.013) and disease-free survival (HR: 3.417, *P* = 0.011). Poor prognosis was found in the non-pCR plus post-nCRT SUVmax ≥ 3 group compared with pCR plus post-nCRT SUVmax < 3 group.

**Conclusions:**

SUVmax ≥ 3 is a poor prognostic factor in esophageal squamous cell carcinoma after trimodality treatment, even in patients having pathological complete response.

## Background

Esophageal cancer (EC) ranks seventh in terms of cancer incidence worldwide in 2020 and sixth in cancer mortality overall. The incidence of esophageal squamous cell carcinoma (ESCC) is high in Asian populations [1]. It has a poor prognosis and high mortality rate because most patients with ESCC are diagnosed at advanced stages.

Patients with locally advanced and potentially curable EC should receive trimodality treatment, which involves neoadjuvant concurrent chemoradiotherapy (nCRT) followed by esophagectomy. With regard to long-term EC outcomes, the CROSS trial found a 38% overall survival (OS) rate in the chemoradiotherapy group, as compared to a 25% OS rate in the surgery-only group in ten years’ follow-up [2]. Studies have shown that nCRT followed by surgery improves survival and local control in locally advanced EC [3–5]. The pathological complete response (pCR) rate of this approach ranges from 13 to 47% [6, 7]. However, previous research has reported a significant fraction of disease recurrence after trimodality treatments [8, 9]. Several risk factors associated with tumor relapse following esophagectomy have been defined, such as poor pathological response to nCRT, positive pathological lymph node status, and lymphovascular space invasion (LVSI) [10–12]. The maximum standard uptake value (SUVmax) and the ratio of change in SUV (ΔSUV ratio) of F-18-fluorodeoxyglucose positron emission tomography/computed tomography (^18^FDG-PET/CT) have also been reported to be useful in predicting the prognosis of EC patients treated with nCRT and subsequent esophagectomy [13, 14].

In this study, we aimed to determine the pathological response in patients with ESCC after trimodality therapy at our hospital and to investigate the prognostic factors associated with SUVmax. We expect that in selected patients with high-risk factors, providing more intensive follow-up and adjuvant treatments could potentially reduce tumor recurrence and prolong patient survival.

## Materials and Methods

### Patients

We retrospectively reviewed the electronic medical records of patients who underwent esophagectomy for clinical stage I–III EC at the Tri-Service General Hospital (TSGH) from January 2011 to December 2018. The initial cohort comprised 93 patients who underwent curative surgery. Of this cohort, four patients diagnosed with adenocarcinoma were excluded. Twelve patients were excluded because they underwent surgery alone without nCRT. Another 12 patients were excluded because they did not undergo post-nCRT ^18^FDG-PET/CT. Fourteen patients having post-operative complications were excluded because the complications affect the survival and have influence on the effect of post-nCRT SUVmax and pathological response. One patient was lost to follow up. Thus, a total of 50 patients who underwent trimodality therapy were eligible for this study.

The staging workup included physical examination, hematologic and biochemistry profiles, contrast-enhanced CT of the chest and abdomen, esophagoscopy, endoscopic ultrasonography (EUS), as well as ^18^FDG-PET/CT before nCRT and 4–6 weeks after nCRT. Flexible bronchoscopy was routinely performed in patients with middle‐third EC to rule out a direct invasion of the trachea-bronchial trees. We used the eighth edition of the American Joint Committee on Cancer Staging Manual in this study.

We extracted baseline information from a prospectively maintained database, including age, sex, body mass index (BMI), tumor histology, tumor differentiation, cT and cN stages of EUS, ycT and ycN stages of EUS, number of resected lymph nodes, ypTNM stage, pathological response, radiotherapy dose, pre- and post-nCRT SUVmax, ΔSUV ratio, LVSI status, extracapsular extension of metastatic lymph nodes, and disease status. Details on recurrence and mortality were obtained from medical records from TSGH and outside hospitals, when available, as well as from documented patient communications. Survival status was documented on the date of the final TSGH clinic visit or by outside communication.

This study was approved by the institutional review board of our hospital (A202105212) on Dec. 24, 2021. The need for patient consent was waived owing to the retrospective study design.

### Treatment and outcomes

Radiotherapy was performed at a daily dose of 1.8–2.0 Gray (Gy) on weekdays for 23–28 fractions. Concurrent chemotherapy was administered intravenously using tri-weekly FP regimen: 75 mg/m^2^ cisplatin on day 1 followed by a 24-h continuous infusion of 800 mg/m^2^ 5-FU for 3 days. Curative esophagectomy with two-field lymph node dissection and reconstruction was performed using cervical esophagogastric anastomosis 4–6 weeks after the completion of nCRT. The surgically resected thoracic ESCC specimens were subjected to routine pathological examination. The pCR was defined as ypT0N0M0 (no residual cancer cells in the resected specimen and no metastatic deposits in the lymph nodes). Patients were followed-up every 3–6 months.

### ^18^FDG-PET/CT protocol

The patients fasted, except for water, 6h before ^18^FDG-PET/CT scanning. Following intravenous injection via the antero-median vein of approximately 10 mCi of ^18^FDG, a whole-body PET/CT scan was performed using the Discovery PET/CT 710 system (GE Healthcare) 60 min after radiotracer injection. Prior to PET imaging acquisition, non-contrast and low-dose spiral CT with 3.75 mm-thickness per slice was performed from head to thigh, and subsequently, reconstructed CT imaging was used to generate the parameters required for PET imaging attenuation correction. Whole-body PET scan was then performed for 25 min. All PET/CT data analysis, including imaging fusion, was performed using Xeleris software (GE Healthcare) according to the standardized operating procedures.

### Statistical analysis

Pearson’s chi-square test was used to compare categorical variables. The student’s *t*‐test was used to compare continuous variables, which were expressed as the mean ± standard deviation. Associations between clinical parameters, OS, and DFS were evaluated by univariate and multivariate analyses using the Cox regression model. The backward method was used to optimize the multivariable model.

Survival curves were plotted using the Kaplan–Meier method, and the significance of differences was tested using log-rank tests. OS was measured from the date of operation until death from any cause or the time of the most recent follow-up. Disease-free survival (DFS) started from the date of operation and continued until death or tumor relapse. All calculations were performed using SPSS version 26.0 (IBM Corp.). We determined an optimal cut-off point for the SUVmax level using the mean value and the receiver operating characteristic (ROC) curves, and the cut-off point was used to compare OS and disease-free survival rates of the patients. The level of statistical significance was set at *P* < 0.05.

## Results

**Fig. 1:**
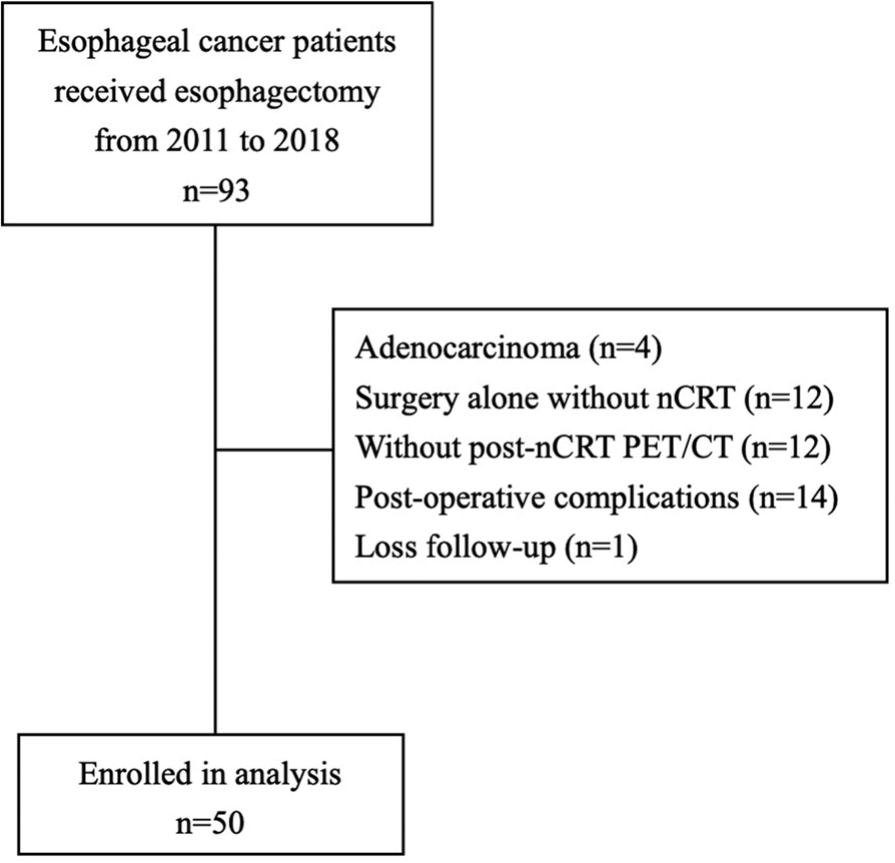
Study design

A total of 93 patients underwent esophagectomy for EC at our hospital between 2011 and 2018. Of this group, 50 patients were included in this study. (Fig. 1.) The demographic data and clinicopathological characteristics of the patients treated with trimodality therapy are listed in Table 1. Our study population comprised 43 men and 7 women, with a mean age of 57.56 ± 7.7 years old, BMI of 21.45 ± 4.08 kg/m^2^, pre-nCRT SUVmax of 12.76 ± 6.59, and post-nCRT SUVmax of 3.79 ± 2.56). Thirty-six (72%) patients had pathological early-stage disease (ypTNM stage I and II), and 14 (28%) patients had pathological advanced-stage disease (ypTNM stage III and IV).

**Table 1:** Characteristics of patients underwent trimodality treatment

Characteristics	Total *n* = 50	Pathological complete response (pCR) *n* = 18	Pathological partial response (pPR) *n* = 32	*P* value
Age (years)		56.67 ± 7.66	58.06 ± 7.79	0.544
Gender				
Male	43	16 (88.9%)	27 (84.4%)	0.505
Female	7	2 (11.1%)	5 (15.6%)	
BMI (kg/m^2^)		23.47 ± 4.16	20.31 ± 3.62	0.007
Differentiation				
Well	2	1 (5.6%)	1 (3.1%)	0.875
Moderate	24	9 (50%)	15 (46.9%)	
Poor	24	8 (44.4%)	16 (50%)	
Post-nCRT EUS T stage				
T1	6	4 (33.3%)	2 (9.5%)	0.107
T2	15	6 (50%)	9 (42.9%)	
T3	12	2 (16.7%)	10 (47.6%)	
Post-nCRT EUS N stage				
N0	26	10 (47.1%)	16 (51.9%)	0.494
N +	7	2 (52.9%)	5 (48.1%)	
ypTNM stage				
I + II (early)	36	18 (100%)	18 (56.3%)	0.001
III + IV (advanced)	14	0 (0%)	14 (43.8%)	
Radiotherapy dose (Gy)		48.53 ± 3.99	48.12 ± 4.80	0.760
Pre-nCRT SUVmax		11.32 ± 4.66	13.57 ± 7.41	0.250
Post-nCRT SUVmax		2.06 ± 1.83	4.76 ± 2.41	< 0.001
< 3	17	11 (61.1%)	6 (18.8%)	0.003
≥ 3	33	7 (38.9%)	26 (81.3%)	
ΔSUV ratio^a^		0.83 ± 0.16	0.48 ± 0.81	0.077
Lymphovascular invasion (LVSI)				
Yes	3	0 (0%)	3 (9.4%)	0.253
No	47	18 (100%)	29 (90.6%)	
Extracapsular extension of metastatic lymph nodes				
Yes	7	0 (0%)	7 (21.9%)	0.034
No	43	18 (100%)	25 (78.1%)	
Numbers of dissected lymph nodes		11.33 ± 5.76	13.27 ± 7.52	0.354

^a^ ΔSUV ratio = (pre-nCRT SUVmax − post-nCRT SUVmax) / pre-nCRT SUVmax

Patients were sorted into two groups: pCR and pathological partial response (pPR), which comprised 18 and 32 patients, respectively. Significant differences were observed in BMI (*P* = 0.007), ypTNM stage (*P* = 0.001), post-nCRT SUVmax (*P* < 0.001), and extracapsular extension of the metastatic lymph nodes (*P* = 0.034) between the two groups.

The potential prognostic factors for OS and DFS estimated using univariate and multivariate analyses are shown in Table 2. The univariate analysis confirmed that the advanced ypTNM stage (hazard ratio [HR]: 2.708, *P* = 0.009), pPR (HR: 3.142, *P* = 0.009), post-nCRT SUVmax ≥ 3 (HR: 5.051, *P* = 0.001), and ΔSUV ratio < 0.8 (HR: 3.748, *P* = 0.031) were poor prognostic factors in regard to OS. In the multivariable analysis, the only independent factor associated with OS was a post-nCRT SUVmax ≥ 3 (HR: 3.665, *P* = 0.013). Univariate analysis showed that advanced ypTNM stage (HR: 2.622, *P* = 0.008), pPR (HR: 3.127, *P* = 0.008), and post-nCRT SUVmax ≥ 3 (HR: 4.165, *P* = 0.002) were poor prognostic factors regarding DFS. In the multivariable analysis, the sole independent factor significantly associated with DFS was a post-nCRT SUVmax ≥ 3 (HR: 3.417, *P* = 0.011). With regard to patients with pPR, post-nCRT SUVmax ≥ 3 was also a poor prognostic factor in OS (HR: 11.416, *P* = 0.004) and DFS (HR: 8.5, *P* = 0.003) (Table 3.)

**Table 2:** Univariate and multivariate Cox regression analyses for overall survival and disease-free survival in esophageal squamous cell carcinoma

	OS				DFS			
Variable	Univariate analysis		Multivariate analysis		Univariate analysis		Multivariate analysis	
	HR (95% CI)	*P* value	HR (95% CI)	*P* value	HR (95% CI)	*P* value	HR (95% CI)	*P* value
Gender (female)	0.832(0.290 ~ 2.387)	0.732			0.617(0.216 ~ 1.761)	0.367		
BMI (< 20 kg/m^2^)	1.666(0.798 ~ 3.477)	0.174			1.732(0.860 ~ 3.488)	0.124		
ypTNM stage (advanced)	2.708(1.276 ~ 5.746)	0.009	1.810(0.773 ~ 4.242)	0.172	2.622(1.284 ~ 5.354)	0.008	1.939(0.874 ~ 4.301)	0.104
Pathological partial response	3.142(1.333 ~ 7.408)	0.009	1.438(0.512 ~ 4.043)	0.490	3.127(1.342 ~ 7.289)	0.008	1.631(0.624 ~ 4.262)	0.318
Pre-nCRT SUVmax (≥ 12)	1.181(0.576 ~ 2.422)	0.650			1.291(0.650 ~ 2.565)	0.466		
Post-nCRT SUVmax (≥ 3)	5.051(1.906 ~ 13.385)	0.001	3.665(1.308 ~ 10.270)	0.013	4.165(1.694 ~ 10.237)	0.002	3.417(1.327 ~ 8.794)	0.011
ΔSUV ratio (< 0.8)	3.748(1.128 ~ 12.454)	0.031	1.559(0.410 ~ 5.925)	0.514	2.399(0.837 ~ 6.878)	0.103

**Table 3:** Multivariate Cox regression analyses for overall survival and disease-free survival in esophageal squamous cell carcinoma with pathological partial response

	OS		DFS	
Variable	HR (95% CI)	*P* value	HR (95% CI)	*P* value
Gender (female)	0.156(0.027 ~ 0.904)	0.038	0.184(0.037 ~ 0.921)	0.039
BMI (< 20 kg/m^2^)	1.001(0.359 ~ 2.793)	0.998	1.136(0.463 ~ 2.788)	0.780
ypTNM stage (advanced)	1.525(0.595 ~ 3.908)	0.380	1.894(0.789 ~ 4.545)	0.153
Pre-nCRT SUVmax (≥ 12)	0.382(0.139 ~ 1.051)	0.062	0.476(0.182 ~ 1.247)	0.131
Post-nCRT SUVmax (≥ 3)	11.416(2.162 ~ 60.284)	0.004	8.500(2.073 ~ 34.853)	0.003
ΔSUV ratio (< 0.8)	0.171(0.010 ~ 2.899)	0.221	0.313(0.021 ~ 4.571)	0.396

**Fig. 2:**
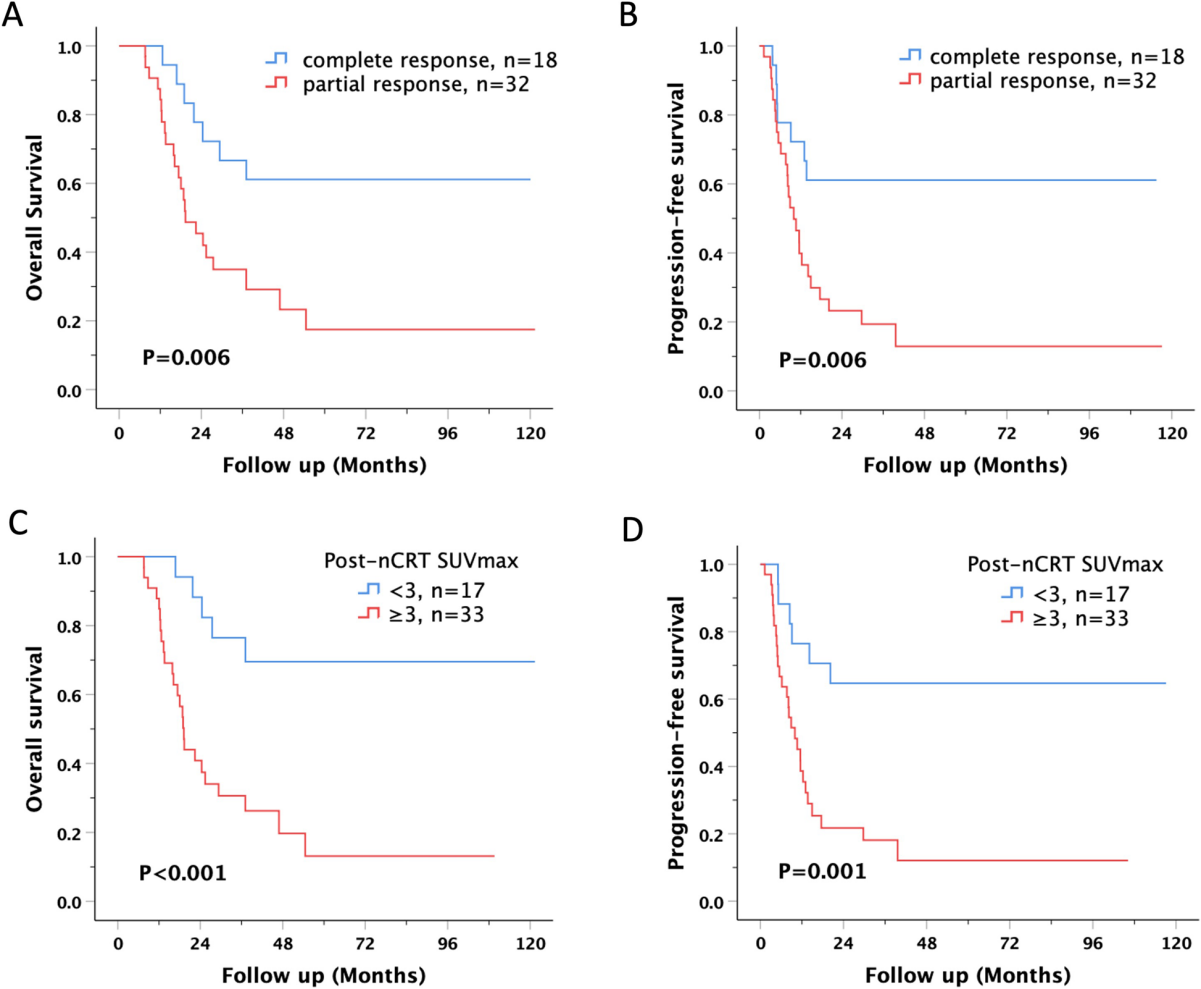
Kaplan–Meier survival curves for overall survival and disease-free survival in esophageal squamous cell carcinoma based on pathological response / post-nCRT SUVmax

**Fig. 3:**
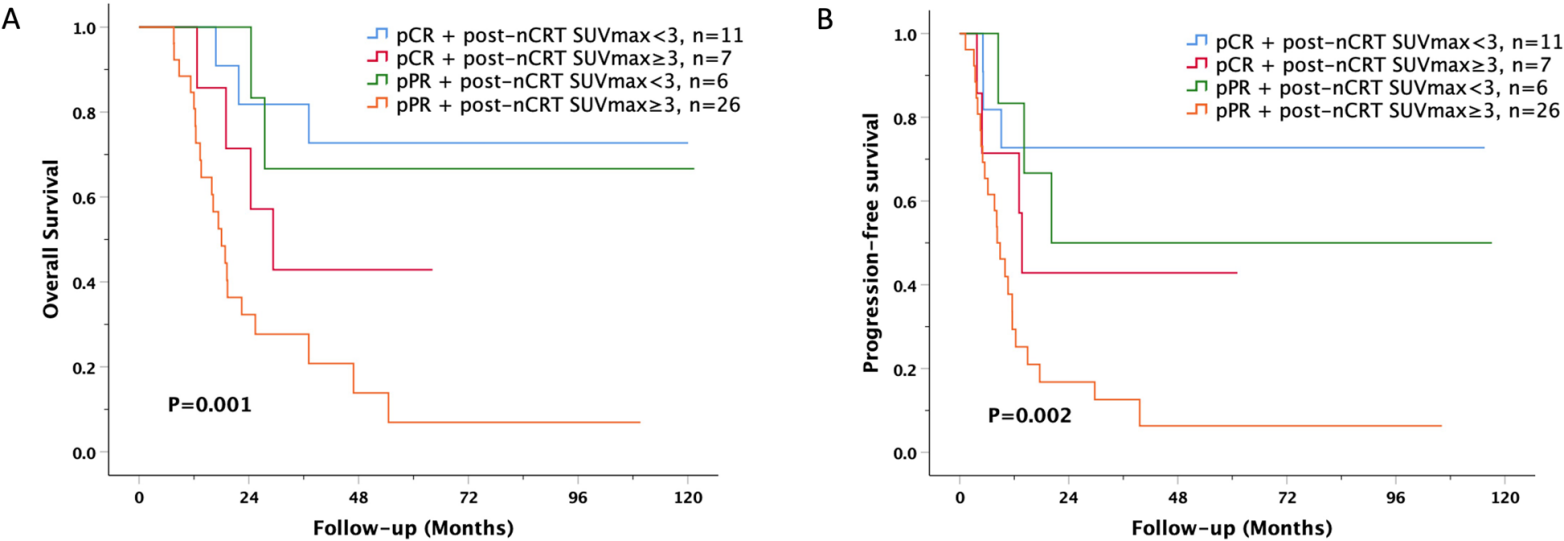
Kaplan–Meier survival curves for overall survival and disease-free survival in esophageal squamous cell carcinoma based on pathological response plus post-nCRT SUVmax

The mean survival periods were 82.33 ± 11.2 months and 39.92 ± 8.0 months in the pCR and pPR groups, respectively. Kaplan–Meier survival analysis was performed, and the OS were 61.1% and 28.1% in the pCR and pPR groups, respectively (*p* = 0.006) (Fig. 2A). The DFS in the pCR group was 61.1% compared with 18.8% in the pPR group (*p* = 0.006)  (Fig. 2B). The OS were 70.6% in the post-nCRT SUVmax < 3 group and 24.2% in the post-nCRT SUVmax ≥ 3 group (*P* < 0.001) (Fig. 2C). The DFS in the post-nCRT SUVmax < 3 group was 64.7% compared with 18.2% in the post-nCRT SUVmax ≥ 3 group (*p* = 0.001) (Fig. 2D). Survival curves based on pathological status and post-nCRT SUVmax are shown in Fig. 3. Patients with a pCR and post-nCRT SUVmax < 3 had the best survival and disease-free rates. Poor prognosis was found in the pPR plus post-nCRT SUVmax ≥ 3 group compared with pCR plus post-nCRT SUVmax < 3 group.

## Discussion

In this study, we proposed an SUVmax of 3.0 in the post-nCRT ^18^FDG-PET/CT as the threshold for predicting prognosis. Changes in SUVmax in the primary tumor are dependent on glucose metabolism and reflect changes in tissue viability in response to chemoradiation. Non-responders are associated with a high risk of local relapse and distant metastasis, and therefore have a worse prognosis. The ΔSUV ratio reflects the response of the tumor tissue after antitumor treatment and seems to be a favorable prognostic factor if it is less than 0.8 in OS.

Among the 17 patients with post-nCRT SUVmax < 3, six patients did not achieve pCR. All these six patients had a positive clinical nodal stage before nCRT, which may indicate that the tumor was relatively more aggressive at the beginning. In addition, two of the six patients had pathological nodal involvement. Okumura et al. showed that patients without pathological lymph node metastasis after nCRT followed by surgery had a good prognosis, even when the effect of nCRT on the primary tumor was poor [15]. There were 7 patients achieved pCR with post-nCRT SUVmax ≥ 3. They seemed to have a poorer prognosis compared with the pPR + post-nCRT SUVmax < 3 group; however, there was no statistically significant difference. Tang et al. found that approximately 75% of patients with ESCC had residual tumors after nCRT and 83.3% of patients without pCR had viable tumor cell residues in the mucosa or submucosa, but in small amounts, whereas the rest had residual tumors in deep layers or lymph nodes [16]. The pathologists did review all the slides of our patients, and the complete response was confirmed. Post-nCRT SUVmax < 3 stand for the lower viability of the tumor cells and the patients with pCR did not have adjuvant treatment and may have circulating tumor cells or occult nodal involvement, which could not be identified in the resected specimen and may explain the poor prognosis.   

Despite medical advances in recent decades, low survival rates and high tumor relapse rates are still common outcomes in EC cases. A more accurate preoperative assessment of treatment response would allow surgeons to make individualized treatment plans for their patients. Van der Wilk et al. reported that patients with EC who achieved clinical complete response after nCRT and underwent active surveillance had a survival outcome comparable to those who underwent surgery immediately after nCRT. In that study, the three-year OS was 77% vs. 55% (*p* = 0.104), and the three-year progression-free survival (PFS) was 60% vs. 54% (*p* = 0.871) in the active surveillance and immediate surgery groups, respectively [17]. Patients who are at high risk of postoperative complications but have a high probability of achieving a pCR could potentially be spared the risks as well as associated potential morbidity and mortality of surgery.

In clinical practice, ^18^FDG-PET/CT can detect glucose metabolic activity, which helps to predict prognosis in several malignancies [18] and is a useful tool as an indicator of residual tumor burden before surgery in ESCC [19]. SUVmax is commonly used as a prognostic parameter in clinical practice because it is easy to measure. Previous studies have found a significant association between early metabolic response and histopathologic tumor regression with SUVmax before and after induction of chemotherapy [20]. In our study, baseline SUVmax did not have any prognostic value. This may be because SUVmax focuses on a single voxel with the highest FDG uptake and thus cannot be used to evaluate the overall metabolic state of the tumor. We also found that post-nCRT SUVmax was helpful for predicting prognosis in terms of DFS and OS, which is consistent with previous studies [21].

The present study has several limitations. First, this was a single-institutional retrospective study, and the sample size was relatively small, which may have led to a large variation in the univariate and multivariate analyses compared to previous studies. Another limitation is the method used for the measurements of metabolic uptake. We could have opted to analyze parameters other than SUVmax, such as metabolic tumor volume (MTV), total lesion glycolysis (TLG), or SUVmean. However, SUVmax is the value that is most often used in our hospital.

In our study, SUVmax ≥ 3 was a poor prognostic factor for ESCC after trimodality treatment, even in patients with a pCR. A consensus has not been reached on the appropriate cut-off value of SUVmax; therefore, further research is warranted.

## Data Availability

The datasets used and/or analysed during the current study are available from the corresponding author on reasonable request.

## Abbreviations

^18^FDG-PET/CT: F-18-fluorodeoxyglucose positron emission tomography/computed tomography
BMI: Body mass index
DFS: Disease-free survival
EC: Esophageal cancer
ESCC: Esophageal squamous cell carcinoma
EUS: Endoscopic ultrasonography
Gy: Gray
HR: Hazard ratio
LVSI: Lymphovascular space invasion
nCRT: Neoadjuvant chemoradiotherapy
OS: Overall survival
pCR: Pathological complete response
PFS: Progression-free survival
pPR: Pathological partial response
ROC: Receiver operating characteristic
SUVmax: The maximum standard uptake value
ΔSUV ratio: The ratio of change in SUV
